# Comparative effectiveness of plant-derived compounds in keloid management: a review

**DOI:** 10.3389/fphar.2025.1576851

**Published:** 2025-07-16

**Authors:** Arya Tjipta Prananda, Sony Eka Nugraha, Putri Cahaya Situmorang, Rony Abdi Syahputra

**Affiliations:** ^1^ Faculty of Medicine, Universitas Sumatera Utara, Medan, Indonesia; ^2^ Department of Pharmaceutical Biology, Faculty of Pharmacy, Universitas Sumatera Utara, Medan, Indonesia; ^3^ Study Program of Biology, Faculty of Mathematic and Natural Science, Universitas Sumatera Utara, Medan, Indonesia; ^4^ Department of Pharmacology, Faculty of Pharmacy, Universitas Sumatera Utara, Medan, Indonesia

**Keywords:** keloid management, plant-derived metabolites, anti-fibrotic therapy, bioavailability challenges, personalized medicine

## Abstract

Keloids are a challenging dermatological condition characterized by excessive scar formation beyond the original wound site, high recurrence rates, and limited treatment efficacy. Current therapies, such as corticosteroids, surgery, and radiotherapy, often yield suboptimal outcomes and adverse effects. This review evaluates the potential of plant-derived metabolites as safer and more effective alternatives for keloid management. Preclinical and clinical studies demonstrate that compounds like curcumin, epigallocatechin gallate (EGCG), and asiaticoside exhibit anti-fibrotic, anti-inflammatory, and antioxidant properties by modulating key pathways (e.g., TGF-β/Smad, NF-κB, and oxidative stress). Espite promising preclinical and early clinical findings, critical challenges hinder the clinical translation of these metabolites. These include poor and variable bioavailability, inconsistencies in extract standardization, and a paucity of large-scale, rigorously designed trials. Moreover, some metabolites may yield conflicting results or exhibit off-target effects in in vitro systems, necessitating caution in interpreting their true therapeutic potential. Future research should focus on optimizing drug delivery systems, conducting large-scale trials, and integrating personalized medicine approaches. Plant-derived metabolites represent a multi-targeted therapeutic strategy with the potential to address unmet needs in keloid treatment.

## 1 Introduction

Keloids are pathological scars characterized by excessive extracellular matrix (ECM) accumulation, particularly collagen, that extends beyond the original injury site and does not regress over time (Tan et al., 2019). Clinically, these lesions present as raised, firm nodules or plaques, often causing pain, pruritus, and cosmetic disfigurement, which can lead to physical and psychological distress. Unlike hypertrophic scars, keloids exhibit persistent growth and fail to resolve, making them challenging to manage (Trace et al., 2016).

The pathogenesis of keloids involves dysregulated wound healing, driven by aberrant fibroblast activity, excessive collagen synthesis, and impaired ECM degradation (Lee et al., 2023). Central to this process is the overexpression of transforming growth factor-beta (TGF-β), which promotes fibroblast-to-myofibroblast differentiation and sustained fibrogenesis. Additional contributors include chronic inflammation mediated by IL-6, TNF-α, and reactive oxygen species (ROS), as well as heightened angiogenesis via vascular endothelial growth factor (VEGF) (Wang et al., 2024). Epidemiologically, keloids disproportionately affect individuals of African, Asian, and Hispanic descent, with incidence rates as high as 20%, likely due to genetic predisposition and skin type (Naik and Farrukh, 2022).

Current therapeutic strategies—such as intralesional corticosteroids, surgical excision, cryotherapy, and radiotherapy—are limited by variable efficacy, high recurrence rates (50%–80%), and adverse effects, including skin atrophy and dyspigmentation (Walsh et al., 2023; Lee and Seol, 2021). These shortcomings underscore the need for novel, targeted therapies that address the underlying molecular mechanisms of keloid formation.

In recent years, plant-derived bioactive metabolites have emerged as promising candidates due to their pleiotropic anti-fibrotic, anti-inflammatory, and antioxidant properties. Preclinical studies have demonstrated that metabolites such as curcumin, epigallocatechin gallate (EGCG), and asiaticoside modulate key pathways in keloid pathogenesis, including TGF-β/Smad signaling, fibroblast proliferation, and oxidative stress (Gowifel et al., 2020; Adamcakova et al., 2023). However, clinical translation faces challenges such as poor bioavailability, lack of standardized formulations, and insufficiently powered trials (Unahabhokha et al., 2015).

This review aims to systematically evaluate the comparative effectiveness of plant-derived metabolites for keloid management. The objectives are threefold: (1) to analyze evidence from preclinical and clinical studies on the mechanisms and therapeutic potential of these metabolites, (2) to compare their efficacy and safety with conventional therapies, and (3) to identify research gaps and propose future directions for optimizing natural product-based therapies. By consolidating existing evidence, this review seeks to provide a comprehensive understanding of the role of plant-derived metabolites in keloid treatment, highlighting their potential as safe and effective alternatives to conventional therapies. Furthermore, it emphasizes the need for further research to address limitations such as bioavailability, formulation standardization, and clinical validation. Advancing our understanding of these metabolites and their mechanisms will pave the way for innovative, multi-targeted therapies that address unmet needs in keloid management and improve patient outcomes.

The inclusion criteria for this review were defined to encompass studies reporting on clinically relevant outcomes, including the reduction of keloid size and scar tissue thickness, the alleviation of subjective symptoms such as itching and pain, and the evaluation of recurrence rates. Eligible studies included those investigating plant-based therapies either as standalone treatments or as adjuvant interventions in combination with conventional approaches such as corticosteroid injections or surgical excision. Furthermore, only studies employing non-invasive methods, such as digital measurement of keloid size and validated clinical assessment tools like the Vancouver Scar Scale (VSS) and the Patient and Observer Scar Assessment Scale (POSAS), were considered. Studies were excluded if they failed to address these primary outcomes, focused solely on invasive methods without incorporating plant-based interventions, or lacked standardized measurement tools or validated clinical scales. Research involving animal models, *in vitro* experiments, or studies with no direct clinical applicability to human populations was also excluded. Additionally, reviews, commentaries, and articles lacking original clinical data were deemed ineligible for inclusion.

## 2 Pathophysiology of keloid formation

### 2.1 Overview of normal wound healing

Wound healing is a complex biological process that occurs in four phases: hemostasis, inflammation, proliferation, and remodeling, where cells and molecules work in coordination to restore tissue integrity (Mamun et al., 2024). The hemostasis phase begins immediately after injury, with platelet activation and aggregation forming a blood clot that provides a temporary scaffold for immune cells and fibroblast migration. In the inflammatory phase, neutrophils and macrophages clear debris and pathogens, while macrophages release pro-inflammatory cytokines (e.g., IL-6, TNF-α) and growth factors such as TGF-β and VEGF to signal the transition to the proliferation phase (Goswami et al., 2021).

During proliferation, fibroblasts produce ECM components, including type III collagen, to form granulation tissue (Olczyk et al., 2014). Endothelial cells, responding to VEGF, initiate angiogenesis to provide oxygen and nutrients to the healing tissue, while keratinocytes migrate to re-epithelialize the wound surface. In the remodeling phase, type III collagen is replaced by type I collagen, which is more stable and stronger. Matrix Metalloproteinases (MMPs) degrade excess ECM, while Tissue Inhibitors of Metalloproteinases (TIMPs) maintain a balance between synthesis and degradation. Proper healing leads to minimal scarring, but disruptions in any phase can result in pathological fibrosis, such as keloids.

### 2.2 Dysregulated mechanisms in keloid pathogenesis

Keloid formation results from dysregulation in wound healing, particularly during the proliferation and remodeling phases, where fibroblasts become hyperactive and collagen production is uncontrolled (Férnandez-Guarino et al., 2024). Keloid fibroblasts exhibit increased proliferative activity and produce excessive type I and III collagen even after healing is complete, leading to scar tissue thickening beyond the original wound site. TGF-β is central to keloid pathogenesis, stimulating fibroblast proliferation, myofibroblast differentiation, ECM synthesis, and inhibiting MMPs that normally degrade excess collagen (Shan et al., 2023). Additionally, VEGF is overexpressed, promoting abnormal angiogenesis that supports fibroblast activity by supplying oxygen and nutrients (Fitzgerald et al., 2018).

**FIGURE 1:**
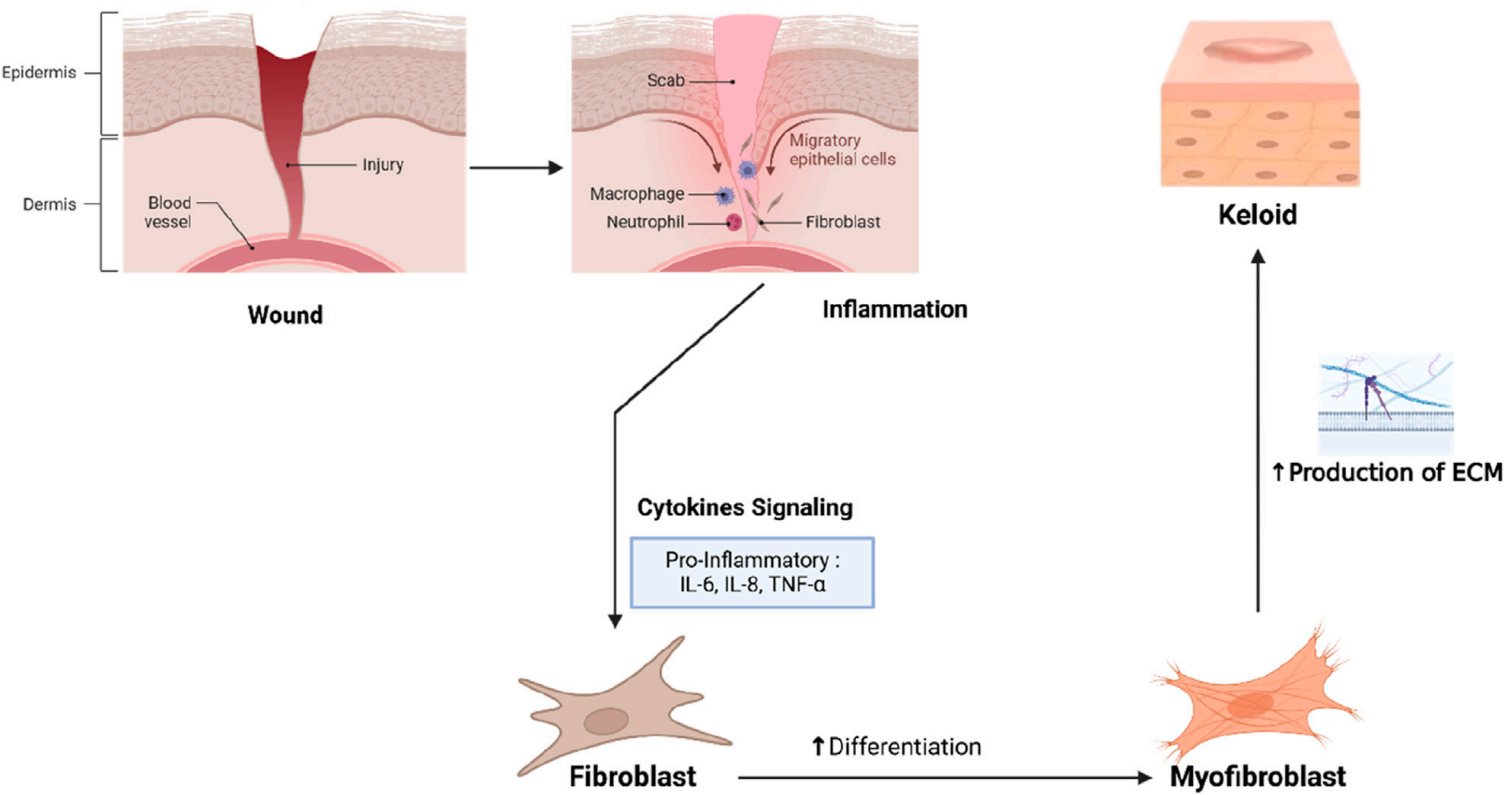
Pathophysiological Mechanism of Keloid Formation.

Pro-inflammatory cytokines like IL-6, TNF-α, and IL-1β sustain chronic inflammation in the keloid tissue, further stimulating fibroblast activity and collagen production (Wang et al., 2020). Oxidative stress, caused by reactive oxygen species (ROS) accumulation, exacerbates fibroblast activation, TGF-β production, and pro-fibrotic signaling, worsening fibrosis (Liu and Desai, 2015). The interplay of fibroblast hyperactivity, TGF-β/VEGF dysregulation, chronic inflammation, and oxidative stress leads to progressive and resilient pathological scarring in keloids (Figure 1).

### 2.3 Need for multi-targeted therapies

Conventional keloid treatments, including corticosteroid injections, surgical excision, cryotherapy, and radiotherapy, are typically single-target approaches that address limited aspects of pathogenesis, such as inflammation or scar size reduction (Walsh et al., 2023). While these treatments may provide temporary relief, keloid recurrence rates remain high (50%–80%), particularly after surgical excision without adequate adjuvant therapy (Lee and Seol, 2021). Furthermore, repeated corticosteroid use can cause adverse effects, such as skin atrophy, telangiectasia, and depigmentation, limiting patient satisfaction. The variability in treatment response reflects the complexity of keloid pathogenesis, involving hyperactive fibroblasts, TGF-β/VEGF dysregulation, and oxidative stress.

This underscores the need for multi-targeted therapies capable of addressing multiple pathogenic pathways simultaneously. Plant-derived metabolites present a promising solution due to their ability to modulate various mechanisms involved in keloid formation. Metabolites such as curcumin, EGCG, and asiaticoside exhibit anti-fibrotic activity by suppressing the TGF-β/Smad pathway, inhibiting fibroblast proliferation, and reducing collagen synthesis (Boo, 2024). They also possess anti-inflammatory and antioxidant properties, mitigating pro-inflammatory cytokine production (e.g., IL-6, TNF-α) and ROS accumulation. Additionally, natural metabolites offer benefits such as safety, bioavailability, and synergistic effects in addressing keloid pathogenesis. Given the multi-targeted nature of these metabolites, they may serve as effective alternatives or complements to conventional therapies. However, further research is required, including validation of their mechanisms of action, preclinical testing, and large-scale clinical trials to assess safety, efficacy, and optimal formulations. Incorporating plant-derived therapies into keloid management could overcome the limitations of conventional approaches, offering a more holistic and innovative solution.

## 3 Plant-derived metabolites

### 3.1 Classification of plant-based metabolites

Plant-based metabolites offer significant therapeutic potential in keloid management due to their ability to target multiple pathogenic pathways, including fibrosis, inflammation, and oxidative stress. These bioactive molecules can be categorized into polyphenols, terpenoids, alkaloids, and glycosides, based on their chemical structure and biological activity. Polyphenols, such as flavonoids (e.g., quercetin and EGCG from green tea) and curcuminoids (e.g., curcumin from Curcuma longa), exhibit strong anti-fibrotic and anti-inflammatory effects. Flavonoids inhibit fibroblast proliferation, suppress pro-fibrotic mediators like TGF-β, and reduce oxidative stress by scavenging free radicals (Chang et al., 2023; Zhao et al., 2024). Curcuminoids reduce ECM deposition and myofibroblast differentiation by modulating the TGF-β/Smad signaling pathway (Xu et al., 2024; Zhang et al., 2020).

Terpenoids, such as asiaticosides from *Centella asiatica* and betulinic acid, regulate fibroblast activity, suppress abnormal angiogenesis, and promote balanced collagen synthesis. Asiaticosides, for instance, reduce VEGF expression and normalize fibroblast behavior, facilitating proper tissue remodeling (Narisepalli et al., 2023; Wang et al., 2024). Alkaloids and glycosides also show promise in inhibiting fibrosis. Berberine, an alkaloid from Berberis, exerts anti-proliferative effects on fibroblasts, reducing excessive collagen production (Bai et al., 2020). Ginsenosides, glycosides from *Panax ginseng*, suppress pro-inflammatory cytokines like IL-6 and TNF-α and lower oxidative stress (Im, 2020). These plant-based metabolites, with their multi-target mechanisms, are ideal candidates for addressing the complex molecular pathways underlying keloid pathogenesis.

### 3.2 Criteria for selecting metabolites in this review

**FIGURE 2:**
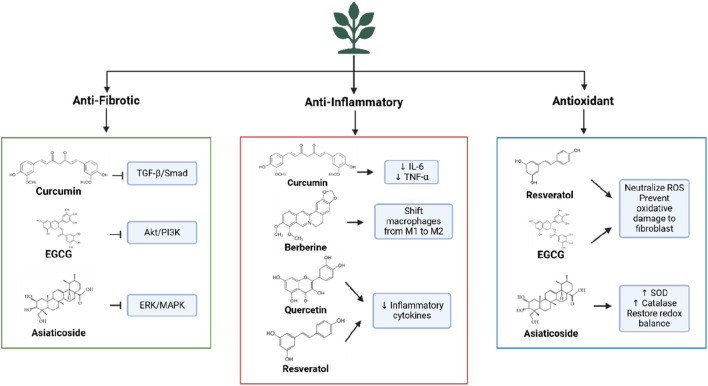
Mechanism of plant-based metabolites in keloid therapy.

The plant-based metabolites discussed in this review were systematically selected based on their therapeutic potential, supported by preclinical studies, clinical evaluations, and mechanistic studies (Figure 2). Priority was given to metabolites with demonstrated efficacy in relevant *in vitro* models (e.g., keloid fibroblast cultures) or *in vivo* models of fibrosis or hypertrophic scarring. Studies needed to provide mechanistic insights, such as inhibition of fibroblast proliferation, regulation of collagen synthesis, or suppression of key pathways (e.g., TGF-β/SMAD, Akt/PI3K, NF-κB) (Hernández-Aquino et al., 2020; Kong et al., 2015; He et al., 2023).

Metabolites supported by clinical data (e.g., randomized controlled trials (RCTs), observational studies, case series) involving patients with keloids or hypertrophic scarring were prioritized. Outcomes of interest included reduction in scar size/thickness, improvement in scar characteristics (e.g., elasticity, color), alleviation of symptoms (e.g., pruritus, pain), recurrence rates, and safety assessments using validated tools (e.g., VSS, POSAS) (Kulawik-Pióro and Goździcka, 2022; Ud-Din et al., 2019).

Metabolites with well-documented anti-fibrotic, anti-inflammatory, and/or antioxidant properties relevant to keloid pathogenesis were included. Evidence of interaction with key molecular pathways (e.g., TGF-β, NF-κB, ROS scavenging) was required. Anti-fibrotic effects were assessed by evaluating inhibition of fibroblast proliferation, suppression of myofibroblast differentiation, and reduction of ECM deposition. Anti-inflammatory activity was measured by modulation of cytokines (e.g., IL-6, TNF-α), and antioxidant activity by the ability to scavenge free radicals and/or enhance endogenous enzymes (e.g., SOD, catalase) (Ashrafizadeh et al., 2020; Huang et al., 2019; Tan et al., 2021).

## 4 Preclinical evidence of plant-derived metabolites

### 4.1 Anti-fibrotic effects

Plant-based metabolites exhibit potent anti-fibrotic effects by targeting multiple molecular pathways involved in keloid pathogenesis. A key pathway is TGF-β/Smad, which regulates fibroblast activation and collagen synthesis (Chen et al., 2015). Preclinical studies show that curcumin inhibits TGF-β1 expression in keloid fibroblasts, disrupting Smad2/3 phosphorylation and preventing pro-fibrotic signal transduction (Murakami and Shigeki, 2024). Curcumin also suppresses fibroblast differentiation into myofibroblasts, which are responsible for tissue contraction and excessive collagen production (Yu et al., 2019). Similarly, EGCG from green tea inhibits the Akt/PI3K and ERK/MAPK pathways, reducing fibroblast proliferation and downregulating collagen genes (COL1A1 and COL3A1), thus limiting ECM deposition (Bae et al., 2018).

Preclinical studies using asiaticosides from *Centella asiatica* show the ability of this metabolite to suppress TGF-β1 expression while inhibiting VEGF formation (He et al., 2023). Additionally, asiaticosides regulate MMPs and TIMPs, which balance collagen degradation and ECM homeostasis, preventing excessive collagen accumulation in keloid tissue (Bian et al., 2013). *In vivo* studies show that asiaticosides reduce scar thickness and improve collagen distribution in fibrosis models (Bian et al., 2013; Yao et al., 2017; Diniz et al., 2023). In summary, plant-based metabolites target key mechanisms involved in fibroblast proliferation, myofibroblast differentiation, and ECM synthesis, with effects on TGF-β, Akt/PI3K, and MMP/TIMP pathways. Their multi-target potential makes them promising candidates for the development of effective anti-keloid therapies.

### 4.2 Anti-inflammatory and immunomodulatory properties

Chronic inflammation plays a critical role in keloid pathogenesis, fostering a microenvironment that promotes fibroblast hyperactivity and excessive ECM production (Nangole and Agak, 2019; Limandjaja et al., 2020). Plant-based metabolites, such as curcumin, have been shown to modulate inflammatory responses by suppressing the NF-κB pathway, a key regulator of pro-inflammatory cytokine expression (Kahkhaie et al., 2019). By inhibiting NF-κB activation, curcumin reduces the levels of cytokines such as IL-6 and TNF-α, decreasing inflammation and preventing fibroblast hyperproliferation in keloid tissue. Additionally, metabolites like berberine can modulate macrophage polarization, which is crucial for resolving inflammation during wound healing. In keloids, an imbalance in macrophage polarization leads to persistent inflammation. Berberine and asiaticosides promote the transition of macrophages from the pro-inflammatory M1 phenotype to the M2 phenotype, reducing pro-fibrotic cytokine production and supporting tissue remodeling (Sun et al., 2023; Xiong et al., 2023).

Other metabolites, including quercetin and resveratrol, also exhibit anti-inflammatory effects by reducing IL-6 and TNF-α levels and decreasing inflammatory cell infiltration in keloid tissue (Song et al., 2024). These mechanisms inhibit fibroblast activation, thereby limiting ECM accumulation. Through their ability to modulate inflammation and immune responses, plant-based metabolites offer a comprehensive approach to addressing the inflammatory components of keloid development.

### 4.3 Antioxidant effects

Oxidative stress, induced by reactive oxygen species (ROS), plays a pivotal role in keloid pathogenesis by enhancing fibroblast activation and exacerbating pro-fibrotic pathways, such as TGF-β. Plant-based metabolites effectively capture ROS and reduce oxidative stress, thereby inhibiting fibroblast hyperactivity and ECM production. Polyphenols like resveratrol and EGCG act as potent antioxidants, neutralizing ROS, protecting fibroblasts from oxidative damage, and attenuating the inflammatory pathways triggered by oxidative stress (Liu et al., 2023).

Moreover, plant-based metabolites can enhance the activity of endogenous antioxidant enzymes, including superoxide dismutase (SOD), catalase, and glutathione peroxidase (GPx). Preclinical studies have shown that asiaticosides increase the expression of SOD and catalase in animal models of fibrosis, promoting redox homeostasis and preventing TGF-β pathway activation due to oxidative stress (Tan et al., 2021). Curcumin has also been reported to reduce ROS production and increase glutathione levels, a key cellular antioxidant (Jakubczyk et al., 2020). By mitigating oxidative stress and enhancing endogenous antioxidant systems, plant-based metabolites protect fibroblasts from oxidative damage and inhibit the activation of pro-fibrotic pathways involved in keloid formation.

### 4.4 Mechanistic insights from in vitro and in vivo studies

*In vitro* and *in vivo* studies provide valuable insights into the mechanisms by which plant-based metabolites inhibit keloid development. *In vitro* studies using keloid fibroblast cultures have demonstrated that metabolites such as curcumin, EGCG, and asiaticoside effectively suppress fibroblast proliferation, inhibit TGF-β1 expression, and reduce collagen synthesis (Ti et al., 2022). Curcumin interferes with the TGF-β/SMAD pathway, while EGCG inhibits the Akt/PI3K pathway, which is involved in myofibroblast differentiation (Liu et al., 2024).

*In vivo* studies using animal models of fibrosis have shown similar results. Mice induced with hypertrophic scar tissue exhibited reduced scar thickness, decreased collagen deposition, and improved ECM organization following treatment with asiaticoside or EGCG (Zhang et al., 2024). Additionally, *in vivo* studies revealed reductions in abnormal angiogenesis, ROS production, and enhancements in antioxidant enzyme activity. A summary of relevant preclinical findings is provided in Table 1.

**TABLE 1:** Preclinical evidence of plant-derived metabolites showing anti-keloid effects.

Plant metabolite	Plant species	Study design	Dose	Target pathway	Mechanism	References
Curcumol	N/A	*In vitro*	160 mg/L of curcumol	Extracellular signal regulated kinase (ERK) signaling	↓ keloid fibroblast proliferation, collagen synthesis, and ERK pathway	Yuan et al. (2021)
Asiaticoside	N/A	*In vivo* (rabbit)	12 and 24 mg/kg/d for 60 days	Transforming growth factor beta (TGF-1)/Smad	↓ hypertrophic scar, ↓ TGF-1, ↑ SMAD7	Huang et al., 2021
Gallic acid	N/A	*In vitro*, *ex vivo* (tissue explant)	25, 50, 100 μM	AKT/ERK	↓ proliferation, migration, invasion, angiogenesis; ↑ apoptosis	Wang et al. (2018)
Madecassoside	*Centella asiatica*	*In vitro*	10, 30, 100 μM for an additional 24 h	PI3K/AKT, p38 mitogen activated protein kinase (MAPK)	↓ migration of keloid fibroblasts	Song et al. (2012)
Asiatic acid	*Centella asiatica*	*In vitro*	3, 10, 30 μM for 24 h	TGF-1/Smad, peroxisome proliferator activated receptor gamma (PPAR-ɤ)	↓ TGF 1-induced collagen, plasminogen activator inhibitor-1 (PAI-1) via PPAR- ɤ	Bian et al. (2013)
Resveratrol	N/A	*In vitro*	20, 40, 80 μM	Hypoxia induciblefactor 1-alpha (HIF-1)	↓ proliferation, ↑ apoptosis, ↓ collagen	Si et al. (2020)
Aspidin PB	*Dryopteris fragrans* (L) Schott	*In vitro*	5, 20 and 40 μmol/L	PI-3K/Akt, Smad	↓ fibrogenesis, collagen, connective tissuegrowth factor (CTGF), alpha-smooth muscleactin (-SMA)	Song et al. (2015)
Genistein	Glycine max	*In vitro*	370, 37, 3.7, 0.37 μM	Nuclear factor kappa-light chain-enhancer of activated B cells (NF-KB), Akt, activator protein 1 (AP-1), TGF	↓ TGF isoforms, CTGF, modulated apoptosis	Jurzak et al., 2014
Wubeizi ointment	*Rhus chinensis* Mill	*In vivo*	3% and 5% Wubeizi ointment	PI3K/AKT, mammalian target of rapamycin (mTOR)	↓ proliferation, ↑ apoptosis, modulated mTOR	Tang et al. (2020a)
Tagitinin C	*Tithonia diversifolia* (Hemsley) A. Gray	*In vitro*	0.125, 0.25, 0.5 and 1 μg/mL	N/A	↓ collagen, ↓ VEGF, no effect on tumor necrosis factor alpha (TNF-α)	Wicaksana et al. (2020)
Corilagin	N/A	*In vitro*	10, 25, 75 μM	TGF-1/Smad	↓ collagen, proliferation, migration, TGF-1	Li et al. (2021)
Galangin	Chinese galangal root	*In vivo*	N/A	TGF-1/Smad	↓ fibrosis, TGF-1, ↑ Smad7	Ru et al. (2021)
Galla chinensis ointment	*Salvia miltiorrhiza*	*In vivo*	5% Galla chinensis ointment	Phosphatase and tensin homolog (PTEN)/Akt/mTOR	↓ keloid, ↑ PTEN/Akt/mTOR, anti-inflammatory	Tang et al. (2020b)

N/A: Not mentioned.

## 5 Clinical evidence of plant-derived metabolites

### 5.1 Overview of clinical studies

A number of clinical studies have evaluated the effectiveness of plant-based metabolites in the management of keloids, including designs such as RCTs, observational studies, and case reports. RCTs, which are considered the gold standard in clinical research, allow for more objective measurements regarding the effectiveness of plant metabolites by comparing intervention and control groups. Observational studies and case reports, despite methodological limitations such as lack of controls or small samples, still make an important contribution to understanding the therapeutic potential of plant-based metabolites in keloid patients.

### 5.2 Effectiveness and safety profiles

Various plant-based metabolites have been evaluated in clinical studies for their effectiveness and safety in managing keloids. Curcumin, derived from *Curcuma longa*, has been tested in topical and nanoparticle formulations. Studies indicate that curcumin reduces keloid thickness, inhibits fibroblast proliferation, and improves scar texture and color by suppressing the TGF-β pathway and reducing oxidative stress and inflammation. Reported side effects, such as mild skin irritation, suggest a favorable safety profile (Murakami and Shigeki, 2024).

*Aloe vera,* known for wound-healing, has been evaluated for keloids. Evidence from Bagheri et al. (2023) suggests *Aloe vera* gel can reduce keloid size, alleviate symptoms like itching and pain, enhance skin hydration, suppress inflammation, and improve scar elasticity, potentially by modulating collagen production. (Bagheri et al., 2023).

*Centella asiatica*, containing asiaticosides, is used in topical formulations. Clinical studies, including Shome et al. (2018), report significant reductions in keloid size, increased elasticity, and improved appearance, attributed to inhibition of TGF-β1, suppression of fibroblast proliferation, and reduction in angiogenesis (Shome et al., 2018).

Green tea polyphenol EGCG shows clinical promise. A study by Ud-Din et al. (2019) found topical EGCG reduced keloid redness, thickness, and inflammatory symptoms. Its mechanisms include suppressing fibroblast activity, inhibiting collagen, and modulating oxidative stress. Comparatively, these metabolites reduce keloid size/thickness and symptoms with minimal side effects like mild irritation, unlike corticosteroids. However, variability in dosage, duration, and formulation complicates cross-study comparison (Ud-Din et al., 2019). A summaries of clinical studies on plant-derived metabolites can be seen on Table 2.

**TABLE 2:** Clinical studies on plant-derived metabolites for keloid treatment.

Treatment type	Study design	Sampel size	Duration	Effectiveness	References
Onion extract gel	Randomized double-blind placebo controlled trial	30	16 weeks	Significant improvements in volume, length, width, induration	Perez et al. (2010)
Allium cepa, allantoin, pentaglycan gel	Open-label nonrandomized controlled clinical trial	30	24 weeks	Significant reduction in erythema and neoangiogenesis in treatment group	Campanati et al., 2010
Onion extract andallantoin patch	Intra individual randomized observer-blind controlledtrial	125	12–24 weeks	Significant improvement in Patient and Observer Scar Assessment Scale, Global Aesthetic Improvement Scale for treated scars	Prager and Gauglitz, 2018
*Copaibaoil in siliconegel*	Randomized double-blind placebo controlled trial	42	84 days	Significant improvement in Manchester Scar Scale, high satisfaction (89%)	Waibel et al., 2021
Aloevera, pracaxioil,epi gallocatechin gallate, carapa oil	Prospective observational survey	522	4–8 months	52%–74% reduction in scar size; 73%–88% reduction in itching	Harris et al., 2018
Epigallocatechin-3-Gallate	Randomizes double-blind trial	62	6 weeks	Effective in reduced keloid redness, thickness, and inflammatory symptoms	Ud-Din et al. (2019)
Aloe vera-based gel cream	Pilot study	42	8 weeks	Effective in reduced keloid size and alleviated symptoms	Bagheri et al. (2023)

## 6 Comparative effectiveness of plant-derived metabolites

### 6.1 Mechanistic comparison

Mechanistic comparisons of plant-based metabolites reveal significant differences in their targeting of molecular pathways, with most metabolites acting in a multi-targeted manner to address keloid pathogenesis. Metabolites like curcumin and EGCG exert anti-fibrotic effects by inhibiting the TGF-β/SMAD pathway, a key mediator of fibroblast activation, myofibroblast differentiation, and collagen synthesis (Ashrafizadeh et al., 2020; Azeredo et al., 2024). Additionally, they suppress pro-inflammatory cytokine production (IL-6, TNF-α) via NF-κB pathway inhibition, reducing the inflammatory environment that promotes keloid growth (Huang et al., 2019; Kang et al., 2024).

Antioxidants like resveratrol, asiaticoside, and EGCG scavenge free radicals and enhance endogenous antioxidants (SOD, catalase), reducing oxidative stress driving fibroblast hyperactivity (Ashraf et al., 2024; Eze et al., 2024; Zheng et al., 2024). This dual action differentiates plant-based metabolites from conventional therapies like corticosteroids, which primarily focus on anti-inflammatory effects but fail to address progressive fibrosis and oxidative stress simultaneously (Choudhury and MacNee, 2017). Thus, plant-based metabolites offer a more effective, holistic, and multi-targeted approach to keloid management by targeting pro-fibrotic, inflammatory, and oxidative pathways (Mushtaq et al., 2023).

### 6.2 Comparative efficacy in preclinical models

Preclinical research using fibroblast and animal models has enabled direct comparisons of the effectiveness of various plant-based metabolites in keloid management. *In vitro* studies show that metabolites such as curcumin, EGCG, and asiaticosides significantly inhibit fibroblast proliferation and myofibroblast differentiation compared to controls (Boo, 2024; Meetam et al., 2024). For example, curcumin and EGCG both suppress TGF-β1 expression and reduce type I and III collagen; however, EGCG demonstrates superior antioxidant effects by inhibiting ROS (Ashrafizadeh et al., 2020; Mokra et al., 2022).

*In vivo* studies on animal models further support these findings. Asiaticosides reduced keloid size, improved collagen matrix organization, and decreased abnormal angiogenesis compared to untreated groups (Bandopadhyay et al., 2023; Song et al., 2024). EGCG was effective in suppressing VEGF production and local inflammation in fibrosis models (Mokra et al., 2022; Zhou et al., 2018). Comparative studies indicated that Asiaticosides may be more effective against angiogenesis, while curcumin excels in modulating TGF-β/Smad and exhibiting antioxidant activity (Pattnaik et al., 2023; Song et al., 2024). These findings highlight the potential of these metabolites to target specific aspects of keloid pathogenesis.

### 6.3 Comparative clinical outcomes

In clinical studies, the effectiveness of plant-based metabolites in managing keloids has been assessed based on clinical outcomes such as keloid size reduction, texture improvement, symptom relief, and recurrence rate. For instance, a study on curcumin reported a 30%–40% reduction in keloid thickness after 8–12 weeks of topical treatment (Di Lorenzo et al., 2024). Epigallocatechin gallate-based formulations significantly improved tissue elasticity and alleviated itching and pain in 70%–80% of patients (Harris et al., 2018). In comparison, Aloe vera demonstrated efficacy in reducing inflammation and enhancing scar hydration, but was less effective than EGCG in inhibiting collagen synthesis (Meetam et al., 2024; Shedoeva et al., 2019). EGCG, derived from green tea polyphenols, effectively reduced redness and keloid thickness with a favorable safety profile, showing no significant side effects (Murakami and Shigeki, 2024; Xu et al., 2021). Overall, epigallocatechin gallate and curcumin were more effective in reducing keloid size and improving elasticity, while EGCG and Aloe vera excelled in managing inflammation and subjective symptoms.

Regarding safety, plant-based metabolites offer significant advantages over conventional treatments such as corticosteroids, with minimal side effects, including mild irritation or local allergic reactions (Wahyuni et al., 2021). However, variability in dosage, formulation, and treatment duration across studies highlights the need for further research with more standardized study designs to allow for more direct comparisons.

### 6.4 Limitations of current comparative studies

Although studies on plant-based metabolites for keloid management show promising results, several significant limitations must be addressed. A primary concern is the heterogeneity in study designs, including variations in measurement methods, treatment duration, and metabolite dosages. For instance, some studies utilized topical formulations, while others employed oral or injectable extracts, which influenced the bioavailability and therapeutic efficacy of the metabolites (Sitarek et al., 2020).

**FIGURE 3:**
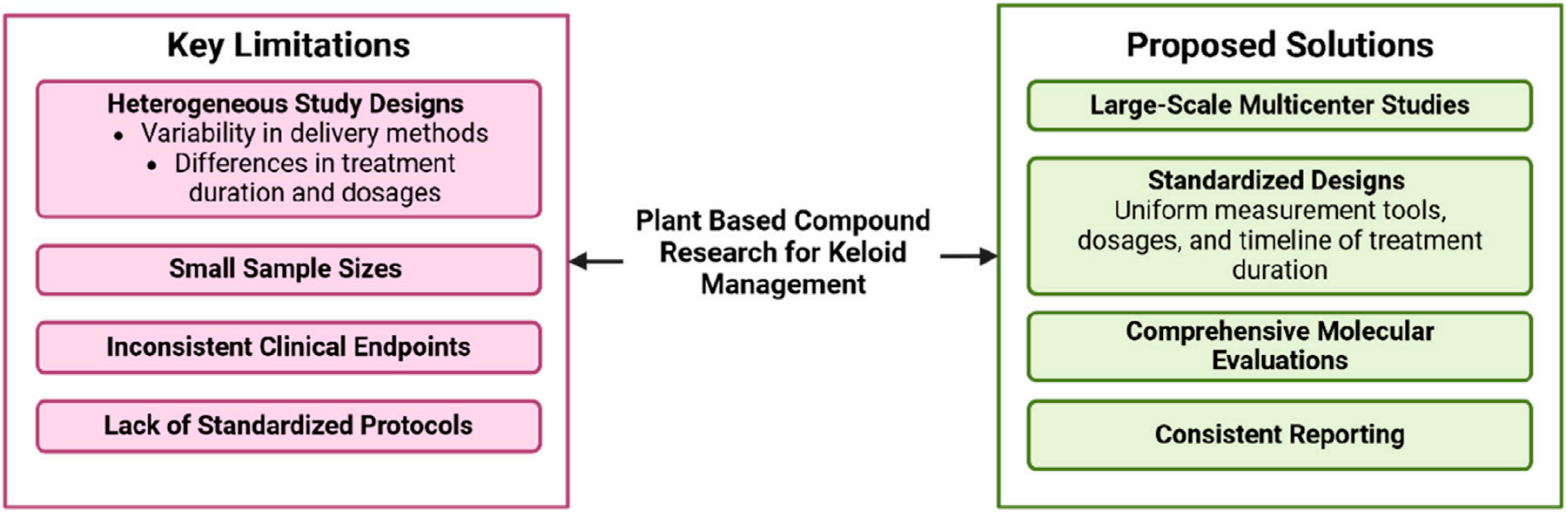
Challenges and future directions in plant-based metanol metabolites research for keloid management.

Additionally, the small sample sizes in many clinical studies limit the ability to draw broad, generalizable conclusions (Figure 3). Variability in clinical endpoints, such as keloid size, recurrence rates, and symptom improvement, further complicates comparisons across studies. Another challenge is the lack of standardized evaluation protocols in both preclinical and clinical research, with inconsistent reporting of key parameters like molecular pathway inhibition, histological changes, and subjective symptom assessments. To address these issues, large-scale, multicenter studies with standardized designs are essential, including uniformity in formulation, dosage, and treatment duration. Comprehensive evaluations utilizing molecular biomarker imaging and analysis technologies will enhance our understanding of the mechanisms of action of plant-based metabolites and ensure more consistent and reliable results.

## 7 Challenges and limitations

Despite the promising potential of plant-based metabolites in keloid management, several challenges and limitations must be addressed to optimize their effectiveness and clinical application. Preclinical studies often face limitations in the standardization of experimental models, both *in vitro* and *in vivo*. *In vitro* fibroblast cultures frequently fail to replicate the complex microenvironment of keloid tissue, hindering the translation of laboratory findings into clinically relevant outcomes (Hofmann et al., 2023). Similarly, existing animal models do not fully mimic the pathophysiological features of human keloids. In clinical studies, small sample sizes, short observation periods, and inadequate control groups undermine the reliability and generalizability of results. Moreover, variability in the purity, formulations, and dosages of metabolites complicates comparisons and consistent interpretations (Dhangar et al., 2017).

Bioavailability and pharmacokinetics remain significant challenges, particularly for metabolites like curcumin, which exhibit low natural bioavailability. Innovations in drug delivery systems, such as nanotechnology or lipid-based formulations, are required to enhance targeted release to keloid tissues (Farasati Far et al., 2023). Additionally, the quality control and reproducibility of plant-based products pose challenges, as variations in extraction methods, raw material sources, and active metabolite concentrations can impact therapeutic efficacy (Upton et al., 2020). Another important limitation is the tendency of many plant-derived metabolites, particularly polyphenols, to interfere with a variety of *in vitro* assays due to their chemical reactivity and non-specific binding properties. These compounds, such as curcumin and EGCG, can act as pan-assay interference compounds (PAINS), leading to assay-dependent effects that may not correspond to true pharmacological activity (Nagle et al., 2006). Their interactions with proteins and redox-sensitive assay components may produce misleading signals, making it challenging to determine specific mechanisms of action. Consequently, some of the therapeutic claims based on *in vitro* data may overestimate their clinical relevance. Future research must apply orthogonal assay systems, validate findings in relevant *in vivo* models, and assess bioavailability and target engagement to distinguish genuine therapeutic effects from assay artifacts. To address these issues, standardization of research protocols is essential, including the development of more representative experimental models, large-scale clinical trials with rigorous designs, and enhanced quality control in product development. Overcoming these obstacles will improve the integration of plant-based therapies into effective, evidence-based keloid treatments.

## 8 Future directions and recommendations

The development of plant compound-based therapies for keloid management requires a more innovative and structured approach. A key focus should be improving the bioavailability and delivery of plant metabolites through advanced technologies such as nanotechnology, which facilitates targeted delivery to keloid tissues. Modern formulation technologies, including liposomes and micelles, can enhance the stability, absorption, and release of active metabolites, particularly those with low bioavailability like curcumin (Obeid et al., 2023; Tabanelli et al., 2021). Additionally, large-scale, rigorously designed clinical trials, such as randomized controlled trials (RCTs), are essential to consistently assess the safety and efficacy of these metabolites. These studies should employ standardized protocols and identify reliable biomarkers to objectively measure therapeutic outcomes.

Further research should explore combination therapies, such as plant metabolites used alongside corticosteroids, radiotherapy, or photobiomodulation therapy (PBMT), to enhance treatment effectiveness and reduce recurrence rates (Chen et al., 2022). The integration of personalized medicine, which tailors treatments based on genetic profiles and molecular characteristics, is a promising direction. This approach allows for individualized therapy adjustments according to keloid features like TGF-β expression, hypoxia, and tissue composition (Morand et al., 2020). With innovations in formulation technology, structured clinical studies, combination therapies, and personalized treatment strategies, plant-based metabolites hold potential as effective, safe, and sustainable solutions for keloid management.

## 9 Conclusion

Plant-based metabolites have demonstrated significant potential in the management of keloids, attributed to their multi-targeted mechanisms, which encompass anti-fibrotic, anti-inflammatory, and antioxidant properties. These metabolites present a notable advantage over conventional therapies, which typically operate through a singular mechanism. The anti-fibrotic effects of metabolites such as curcumin, asiaticoside, and EGCG are mediated through the inhibition of the TGF-β/SMAD signaling pathway, suppression of fibroblast proliferation, and reduction in collagen synthesis. Furthermore, the anti-inflammatory activity of these metabolites is exerted by the downregulation of pro-inflammatory cytokines, such as IL-6 and TNF-α. Additionally, the antioxidant properties of polyphenols like resveratrol and EGCG help mitigate oxidative stress, a critical factor in fibroblast hyperactivity and the accumulation of extracellular matrix components in keloids. Notwithstanding the promising therapeutic effects of these plant-based metabolites, there exists an imperative need for further investigation aimed at standardizing treatment parameters, including dosage, formulation, and treatment duration. Establishing clear and evidence-based clinical guidelines is essential for the consistent and effective application of these therapies in clinical practice. To substantiate their clinical utility, large-scale, rigorously designed randomized controlled trials (RCTs) are required to comprehensively assess the safety, efficacy, and long-term outcomes of these metabolites. In parallel, optimization of delivery systems, such as nanoparticles, liposomes, and micelles, should be explored to enhance the bioavailability and stability of plant-based metabolites, particularly those with inherent pharmacokinetic limitations.

The incorporation of personalized medicine strategies represents an important avenue for future research. Tailoring therapeutic interventions based on individual patients' genetic profiles and keloid characteristics could not only enhance treatment efficacy but also reduce the risk of recurrence. Personalized approaches could enable the identification of the most suitable plant-based metabolites for specific patient populations, thus improving overall clinical outcomes. In conclusion, with continued research efforts focused on standardization, optimization of drug delivery systems, and the integration of personalized medicine, plant-based metabolites hold considerable promise as innovative, effective, and sustainable solutions for keloid management. These metabolites offer a valuable alternative to conventional therapies, with the potential to provide more targeted, safe, and enduring treatments for individuals suffering from this complex dermatological condition.
